# Traditional Chinese medicine treatment in a patient with severe COVID-19: A case report

**DOI:** 10.1097/MD.0000000000050149

**Published:** 2026-08-28

**Authors:** Ming Zhong, Shiqi Sun, Fengjun Zhang, Wei Zhang

**Affiliations:** a Department of Critical Care Medicine, Ruijin Hospital, School of Medicine, Shanghai Jiaotong University, Shanghai, China; b Department of Pulmonary Diseases, Shuguang Hospital Affiliated to Shanghai University of Traditional Chinese Medicine, Shanghai, China; c Department of Pulmonary Diseases, Shuguang Hospital, Shanghai Institute of Infectious Diseases and Biosecurity, Shanghai University of Traditional Chinese Medicine, Shanghai, China; d Zhang Wei Baoshan famous traditional Chinese medicine inheritance studio, Shanghai Baoshan Hospital of Integrated Chinese and Western Medicine, Shanghai, China; e Institute of Infectious Diseases, Shanghai Institute of Traditional Chinese Medicine, Shanghai, China.

**Keywords:** integrative Chinese and Western medicine (ICWM), persistent inflammation–immunosuppression–catabolism syndrome (PICS), respiratory failure, severe COVID-19 infection, traditional Chinese medicine (TCM)

## Abstract

**Rationale:**

Elderly patients with severe COVID-19 frequently develop chronic critical illness marked by prolonged ventilation, multidrug-resistant infections, enteral-nutrition (EN) intolerance, and persistent inflammation–immunosuppression–catabolism syndrome, making liberation from the ventilator and recovery difficult. Integrative Chinese and Western medicine (ICWM) is hypothesized to modulate immunity and the gut–lung axis and to stabilize frail physiology during long ICU courses.

**Patient concerns:**

An 87-year-old man with >10-year Parkinson’s disease experienced septic shock, invasive ventilation, and tracheotomy after COVID-19, prompting a request for ICWM.

**Diagnoses:**

Severe pneumonia due to COVID-19 (CURB-65 score of 3); multiple organ dysfunction syndrome (respiratory failure, acute renal failure, and acute heart failure); sepsis; malnutrition; Parkinson’s disease; and lower extremity venous thrombosis. The traditional Chinese medicine diagnosis was based on “epidemic disease,” with dampness toxin stagnation in the lung, and spleen and kidney deficiency.

**Interventions:**

A multidisciplinary team delivered individualized ICWM aligned with the “Shanghai Expert Consensus” and the traditional Chinese medicine principle of “benefiting qi and strengthening the body.” Prescriptions were dynamically adjusted to syndrome evolution and to gastrointestinal tolerance, successively including Buzhong Yiqi decoction, Shenling Baizhu powder, Weijing decoction, Tounong decoction, and Shengxian decoction; when gastrointestinal absorption was limited or shock was present, intravenous Shenfu injection was added. Standard care comprised culture-guided antimicrobials, bronchoscopy-assisted secretion clearance, invasive ventilatory support, and nutrition therapy with EN. The ICWM plan was titrated specifically to address EN-intolerance diarrhea and to support nutritional rehabilitation.

**Outcomes:**

After treatment, intolerance symptoms improved; sputum cultures converted to negative by ICU day 41. The patient weaned from mechanical ventilation and the tracheal tube 10 days later, and remained afebrile without productive cough, enabling discharge to home convalescence.

**Lessons:**

This case highlights the need for ICWM in critically ill patients, especially in those with chronic critical illness and persistent inflammation–immunosuppression–catabolism syndrome.

## 1. Introduction

COVID-19 is characterized primarily by symptoms such as fever, fatigue, and cough. Some patients may experience severe conditions, in which they develop hypoxemia, respiratory failure, acute respiratory distress syndrome (ARDS), and septic shock. In particular, elderly patients often have a deficiency in organ function reserves and general frailty, which can lead to rapid deterioration of their condition after infection. Traditional Chinese medicine (TCM) has demonstrated desirable therapeutic effects in the treatment of severe COVID-19. In this case, we report the treatment of a patient in accordance with the “Shanghai Expert Consensus on Traditional Chinese Medicine Treatment for Elderly Patients with COVID-19 Infection,”^[1]^ guided by the theory of traditional Chinese medicine to improve physical resistance, which was achieved by strengthening immune function (manifesting as, for example, greater lymphocyte counts), improving the gastrointestinal barrier (e.g., reducing intestinal microbiota translocation), and suppressing excessive inflammation (e.g., lowering CRP and PCT levels) to restore the body’s balance.^[2]^ This case highlights the synergistic effects of TCM and modern medicine in the management of critical illnesses.

## 2. Case description

The patient was a 87-year-old man admitted to our hospital on August 9, 2023, with a chief complaint of recurrent cough and fever for 2 months, which had worsened in the prior 5 days.

The patient’s history was as follows: the patient had experienced coughing with phlegm, chest tightness, shortness of breath, and palpitations without any clear cause 2 months ago. He denied chest pain, radiating pain, cold sweats, dizziness, acid reflux, or heartburn. On June 4, he experienced shortness of breath and sweating, with oxygen saturation fluctuating between 78% and 90%. Reverse transcriptase–polymerase chain reaction (RT–PCR) of a sample from a pharyngeal swab was positive for SARS-CoV-2 on January 29. Therefore, the patient was diagnosed with COVID-19. His treatment included noninvasive ventilation (NIV) support, antiinflammatory and antiviral therapies, and symptomatic management with expectorant and cough suppressant agents. Despite these interventions, his dyspnea progressively worsened, and he soon developed shock. Vasopressors (dopamine and norepinephrine) were administered to maintain blood pressure on July 13, and he underwent tracheal intubation and invasive ventilation. A tracheotomy was performed on July 20. During this period, bedside bronchoscopy was performed for sputum clearance. Blood culture revealed *Proteus mirabilis* and *Candida albicans*. Sputum culture revealed *Klebsiella pneumoniae*, *Pseudomonas aeruginosa*, and *P. mirabilis*. The anti-infection regimen consisted of ceftazidime/avibactam (Q12h) + posaconazole (Q12h) + metronidazole (Q12h). After antibiotic treatment, the patient’s symptoms improved, and the ventilator parameters were decreased. However, his fever recurred quickly, and his general condition continued to deteriorate. Thus, the demand for ventilator support increased. The patient’s antibiotic therapy was adjusted according to the results of the sputum culture to include polymyxin, morinidazole, and meropenem. Given that the patient’s infection remained unstable and difficult to control despite standard treatment at a tertiary hospital, the patient’s family sought TCM as adjunctive therapy. Consequently, the patient was transferred to our ICU.

His medical history included Parkinson’s disease for more than 10 years, with metoba tablets 1# qd to control the symptoms. He denied a history of hypertension, diabetes, heart disease, other internal diseases, hepatitis, tuberculosis, other infectious diseases, or surgical or major trauma.

On physician examination, the patient was confused and had an altered mental status (Glasgow Coma Scale (GCS) score: E4 Vt M5) and showed signs of emaciation and cachexia with temporal wasting (an NRS-2002 score of 6, indicating severe malnutrition). The BP was 123/91 mm Hg, the temperature was 36.5°C, and the HR was 68 bpm. The patient underwent tracheotomy and mechanical ventilation with the following settings: P-SIMV mode, FiO_2_ 50%, f 5 bpm, PSV 12 cm H_2_O, PEEP 6 cm H_2_O, and PCV 10 cm H_2_O. The APACHE II score was 16; the SOFA score was 7.

## 3. Diagnosis

The patient was diagnosed with the following: 1) severe pneumonia (COVID-19; CURB-65 score: 3 points); 2) multiple organ dysfunction syndrome (type II respiratory failure, acute heart failure, and acute renal failure); 3) sepsis; 4) malnutrition; 5) Parkinson’s disease; and 6) lower extremity venous thrombosis.

The TCM diagnosis was epidemic disease, dampness toxin stagnation lung, and “spleen” and kidney deficiency.

## 4. Therapeutic interventions

### 4.1. General treatment

**Figure 1.**
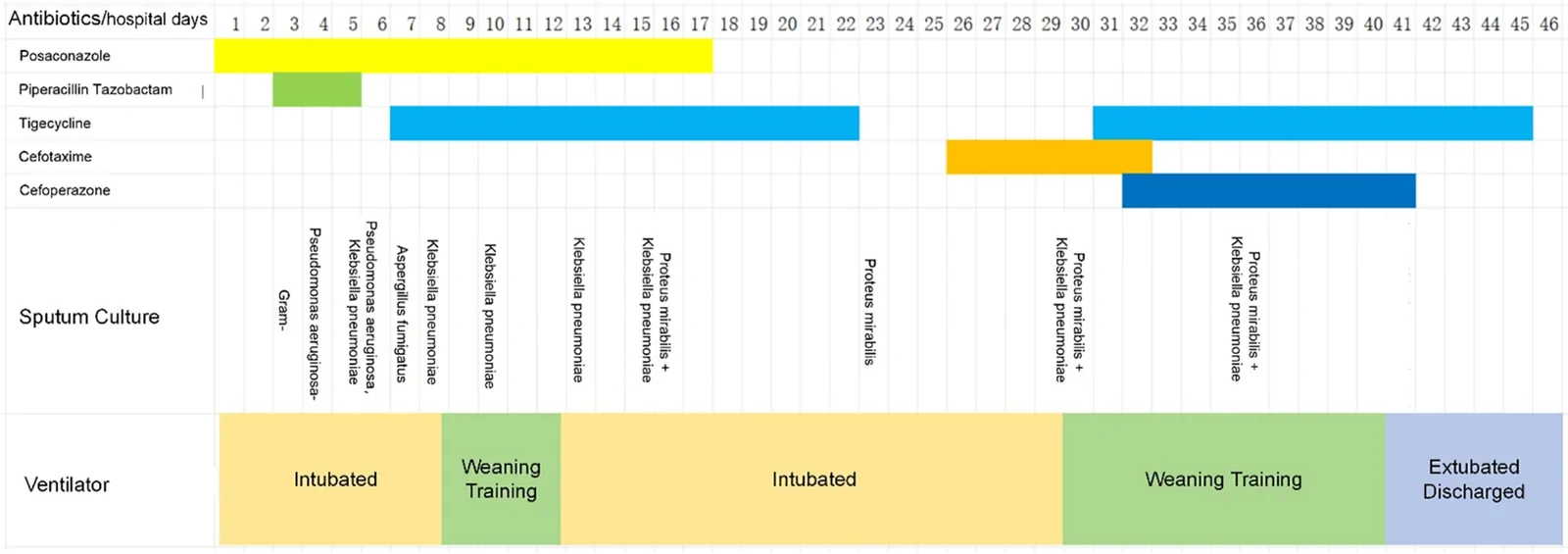
Antibiotic and ventilator use timeline in this case.

Following standard treatment, the patient received invasive mechanical ventilation, antibiotic treatment, and nutritional support (enteral nutrition), as shown in Figure 1.

### 4.2. TCM treatment

The TCM team visited the patient daily and developed prescriptions on the basis of TCM syndrome differentiation. In succession, the patient was given Buzhong Yiqi decoction (补中益气汤), Shenling Baizhu powder (参苓白术散), Weijing decoction (千金苇茎汤), Tounong decoction (透脓汤), Shengxian decoction (升陷汤), and other combinations. Shengfu injections (参附注射液) were also used intravenously.

The core TCM strategy was “benefitting qi and strengthening the body” (益气扶正 in Chinese), which means strengthening the body’s resistance, together with a flexible combination of other strategies, such as invigorating the spleen to eliminate diarrhea (健脾止泻), dissipating phlegm to discharge pus (化湿透脓), restoring Yang to reduce energy loss (回阳固脱) (the patient was in shock as vasopressors were used), and activating blood to promote water removal (活血利水). The TCM prescriptions and clinical features are listed in Table 1 and Table S1, Supplemental Digital Content 1.

**Table 1:** Patient’s blood test report and TCM prescriptions.

Date	WBC	CRP	PCT	Cr	PaO_2_/FiO_2_	Albumin	Globulin	defecating frequency	Alb injection	Total input	Total Output	EN	TCM prescription
8/9	6.7	12.33	2.86	117.8	103.5			1	20g	1100	1150	Total protein formulation (TPF) 1000Kcal [contain protein (pro) 40g]	Combination: Buzhong Yiqi Decoction, Shenling Baizhu Powder, Weijing Decoction, Tounong Powder
8/10					312.5	37.5	22.7	6	20g	2434	1800	TPF 1500Kcal (pro 60g)	
8/14	3.24	90.59	0.85	106.3	332.5	33.7	22.6	6	20g	2100	1850	SP 1000Kcal (pro 40g)	Combination: Shenling Baizhu Powder, Buzhong Yiqi Decoction and Shengxian Decoction Shenfu Injection 40ml q12h
8/16	3.08	91.42	2.61	101		35.2	21.6	10	20g	1805	1800	SP 1000Kcal (pro 40g)	Combination: Buzhong Yiqi Decoction, Shenling Baizhu Powder, Xiangsha Liujunzi Decoction Shenfu Injection 40ml q12h
8/28	4.15	32.44	1.42	57.1	362.5	31.2	20	6	20g	2141	4050	TPF 1500Kcal + whey 70g	
9/4								1	20g	2011	1700	TPF 2000Kcal + whey 70g	Combination: Shenling Baizhu Powder, Jianpi Lizhong Decoction
9/5	8.36	122.57	1.58	72.3	380	42.2	22.4	1	20g	1828	1300	TPF 2000Kcal + whey 70g	
9/8	15.12	295.39	1.35	73.7		39.9	23.7	2	20g	1274	1200	TPF 2450Kcal + Whey 80g	
9/12	17.03	111.26	0.54	67	472.5	39.7	26.9	5	20g	1756	1800	TPF 2000Kcal + pro 75g	
9/24	9.04			51.2		36.7	23.3	1	20g	1550	2400	TPF 1500ml pro 75g	

Cr = creatinine, CRP = C-reactive protein, EN = enteral nutrition, PaO2/FiO2 = arterial oxygen partial pressure/fraction of inspired oxygen, PCT = procalcitonin, WBC = white blood cell count.

### 4.3. Follow-up and outcome

The patient received mechanical ventilation for > 2 months. He experienced recurrent resistant bacterial infections, including infections with carbapenem-resistant *K. pneumoniae* (CRKP), carbapenem-resistant *P. aeruginosa* (CRPA), extended-spectrum beta-lactamase (ESBL)-producing bacteria, and *P. mirabilis*. The sputum culture test results were negative on the 41^st^ ICU day.

He also developed intolerance to enteral nutrition, which presented as diarrhea with watery stool and frequent bowel movement. The symptoms of intolerance were gradually alleviated after the administration of TCM.

He was weaned off the ventilator on September 12, and the tracheal tube was removed 10 days later. The patient’s condition was stable for 1 week, with no obvious coughing or phlegm production and a stable body temperature. The patient was discharged and recovered at home.

## 5. Discussion

Although antivirals have been widely used, especially for treating COVID-19, the overall outcomes remain poor among elderly patients.^[3]^ In addition, adverse effects associated with these drugs are more frequent in elderly patients.^[4,5]^ TCM has played an important role and shown excellent results in treating COVID-19 in China^[6,7]^ as well as in the overall treatment of critically ill patients.

In this report, we present the case of an elderly male who developed severe pneumonia and respiratory failure after contracting COVID-19. After he received standard therapy, such as invasive mechanical ventilation and antiviral, antibiotic, and other supportive treatments, his condition fluctuated, and the patient was considered unstable. He failed to be weaned off the ventilator, developed recurrent resistant bacterial infections, and exhibited intolerance to enteral nutrition. Notably, several other aspects of his condition further complicated the treatment. First, hospitalized patients are particularly susceptible to various infections, especially those caused by multidrug-resistant organisms (MDROs). The association between COVID-19 and the incidence of MDRO infections, combined with a prolonged ICU stay and extended antibiotic regimens, significantly increases the risk of such infections.^[8]^ Additionally, motor disorders, such as Parkinson’s disease, can impair a patient’s ability to effectively clear sputum, further complicating pathogen elimination.^[9]^ Second, intolerance to enteral nutrition (EN) results in malabsorption of the EN, further exacerbating nutrient deficiency and weight loss.^[10]^ This contributes to ICU-acquired weakness,^[11]^ thereby further delaying ventilator weaning. Third, the patient exhibited a chronic critical illness that was characterized by recurrent infections, leading to the development of persistent inflammation, immunosuppression, and catabolism syndrome (PICS).^[12]^ This condition further prolonged the patient’s recovery.

MDRO infections and prolonged antibiotic therapy may lead to diminished intestinal microbiota, resulting in EN intolerance and malnutrition that further impair immunity and increase the risk of recurrent MDRO infections, ultimately contributing to the development of PICS. To break this vicious cycle, it is important to maintain the patient’s intestinal function, achieve adequate nutrition, and strengthen the patient’s immune function are important to eliminate the MDROs. Gastrointestinal malfunction and PICS are increasingly recognized as poor prognostic indicators in critically ill patients with limited clinical management options. Traditional Chinese medicine has opened up new treatment options for these patients.

Diarrhoea is a common symptom of intolerance in ICU patients receiving enteral nutrition. Independent risk factors for its occurrence include age, tracheostomy, antibiotic use, and the dose of enteral nutrition.^[13–15]^ According to a previous study,^[15]^ Shenling Baizhu powder can improve the structure of the intestinal microbiota, shorten the time required to stop the diarrhea and mechanical ventilation, preserve the function of the intestine, ensure the smooth implementation of enteral nutrition (thus improving the nutritional status of the patient), and establishing a foundation for successful ventilator weaning later in the treatment.

With TCM treatment, the patient’s “Pi” (“spleen”) and “Wei” (“stomach”) functions were optimized to facilitate the effective implementation of enteral nutrition. Buzhong Yiqi decoction and Baizhu powder (参苓白术散)^[16]^ are common traditional Chinese medicine prescriptions used to regulate the functions of the spleen and stomach, aiming to improve digestion and absorption.. These prescriptions contain ginseng, astragalus, and angelica, which may be beneficial for increasing energy in TCM. Studies have also shown that ginseng and *Astragalus* improve immunity and regulate glycerophospholipid metabolism, leading to increases in the spleen and thymus indices, proliferation of splenic lymphocytes, and an increase in the cytotoxic activity of NK cells.^[17]^ Huang et al reported that ginseng polysaccharides altered the gut microbiota and the kynurenine/tryptophan ratio, which may help reshape the immune system.^[18]^ Ginseng-derived nanoparticles have been shown to reprogram macrophages, thereby ameliorating T-cell exhaustion.^[19]^ Other ingredients in these formulations, such as *Scutellaria baicalensis*, *Glycyrrhiza uralensis*,^[20]^ and *Atractylodes macrocephala*,^[21]^ can alleviate diarrhea.

Moreover, the Weijing decoction and Shengxian decoction are commonly used combinations in TCM for managing infections. Research has demonstrated that the constituents of these formulations, such as the thorns of soapberry, reed root, turmeric,^[21]^ and rhubarb,^[22]^ possess antibiotic-like properties.

However, the gastrointestinal (GI) system is frequently compromised during critical illness,^[23]^ potentially leading to malabsorption of TCM formulations. In such cases, intravenous administration of TCM-based injections, such as Xuebijing (XBJ) and Shenfu injection (SFIs), remains viable options. Clinical and experimental studies have consistently demonstrated the efficacy of SFIs in critical care settings.^[24,25]^ In patients with sepsis, the levels of both ET-1 and CXCL8, both risk factors for heart failure in these patients, were significantly increased following SFI administration.^[26]^ Shenfu injections may inhibit PI3K/Akt-mediated glycolysis, thereby alleviating endotoxaemia-induced endothelial dysfunction (such as by suppressing ET-1 upregulation) and reducing organ injury.^[25,27]^ Furthermore, Shenfu injections play a pivotal role in shortening the duration of vasopressor use, promoting microcirculation in the GI tract, and improving GI function.^[28]^ Similarly, XBJ injections have been associated with reduced mortality in patients with sepsis. Mechanistically. XBJ injection may inhibit the ACLY/MYB/RIG-I axis by ameliorating vascular endothelial dysfunction.^[29]^ Both XBJ and SFI are derived from well-established TCM formulas, offering a reliable and effective approach to integrating TCM into modern ICU practices,^[30,31]^ even in the presence of potential GI dysfunction during critical illness.

This case also exemplifies the characteristics of Shanghai-style TCM, which emphasizes gentle and light medication.^[32]^ The dosage of each ingredient was primarily 6 to 9 g/day, which falls within the recommended range according to the Chinese Pharmacopoeia. This approach is conducive to minimizing the potential adverse effects of medications.

However, this report is a single-case study, and the results may be influenced by concurrent adjustments to antibiotic therapy and optimized ventilator management. Moreover, the placebo effect cannot be ruled out, and further validation through large-scale studies is needed.

## 6. Conclusion

We present a case of an elderly patient who was successfully treated with a combination of TCM and standard care (integrative Chinese and Western medicine), allowing recovery from COVID-19 infection. This case highlights the need for integrative medicine in critically ill patients, especially those with chronic critical illness and PICS.

## Author contributions

**Conceptualization:** Ming Zhong, Shiqi Sun, Fengjun Zhang, Wei Zhang.

**Data curation:** Ming Zhong, Shiqi Sun, Wei Zhang.

**Formal analysis:** Ming Zhong, Shiqi Sun.

**Investigation:** Ming Zhong.

**Validation:** Ming Zhong.

**Visualization:** Ming Zhong.

**Writing – original draft:** Ming Zhong, Shiqi Sun.

**Writing – review & editing:** Ming Zhong, Fengjun Zhang, Wei Zhang.

**Funding acquisition:** Shiqi Sun, Wei Zhang.

**Resources:** Fengjun Zhang, Wei Zhang.

**Supervision:** Fengjun Zhang, Wei Zhang.

## Correction

This article originally published with an incorrect corresponding author Wei Zhang’s affiliation has now been corrected online with the following details “Department of Pulmonary Diseases, Shuguang Hospital, Shanghai Institute of Infectious Diseases and Biosecurity, Shanghai University of Traditional Chinese Medicine, Shanghai, China (e-mail: zhangw1190@sina.com).”


