# Supplementary material for: Traditional Chinese medicine treatment in a patient with severe COVID-19: A case report
Source: Medicine (Baltimore). 2026 Aug 28;105(35):e50149. doi: 10.1097/MD.0000000000050149 (PMC13529106; doi:10.1097/MD.0000000000050149)
Supplement: Supplementary file 1 [file medi-105-e50149-s001.docx]

**Supplementary Table S1** Dosage of traditional Chinese medicine classic prescriptions.

| **Name** | **Drug name and dosage** |
| --- | --- |
| Buzhong Yiqi Decoction | Raw Astragalus (Shenghuangqi) 15g, Fried White Atractylodes (Chaobaizhu) 12g, Angelica Sinensis (Danggui) 10g, Ginseng (Renshen) 6g, Licorice (Gancao) 10g, Bupleurum chinense (Chaihu) 10g, Cimicifuga (Shengma) 10g, Ginger (Shengjiang)10g, Jujube (Dazao) 10g |
| Shenling Baizhu Powder | Dolichos lablab (Baibiandou) 30g, Poria (Fuling) 20g, Raw Chinese Yam (Shengshanyao) 20g, Ginseng (Renshen) 6g, Bran-Fried Atractylodes (Fuchaobaizhu) 15g, Lotus Seed (Lianzi) 30g, Amomum Villosum (Sharen) 6g, Honey-Fried Licorice (Zhigancao) 10g, Raw Coix Seed (Shengyiyiren) 30g, Platycodon Root (Jiegeng)10g |
| Weijing Decoction | Reed Rhizome (Lugen) 30g, Raw Coix Seed (Shengyiyiren) 30g, Peach Kernel (Taoren) 15g, Fried Winter Melon Seed (Chaodongguazi) 30g |
| Tounong Powder | Astragalus Root (Huangqi) 30g, Chinese Honeylocust Spine (Zaojiaoci) 6g, Dahurian Angelica Root (Baizhi) 10g, Chuanxiong Rhizome (Sichuan Lovage) 10g, Fried Burdock Seed (Arctii Fructus) 10g, Japanese Honeysuckle Flower Bud (Jinyinhua) 15g, Chinese Angelica Root (Dang Gui) 12g |
| Xiangsha Liujunzi Decoction | Ginseng (Renshen) 6g, Bran-Fried Atractylodes (Fuchaobaizhu) 15g, Poria (Fuling) 20g, Honey-Fried Licorice (Zhigancao) 10g, Processed Pinellia (Zhibanxia) 6g, Dried Tangerine Peel (Chenpi) 15g, Amomum Villosum (Sharen) 6g, Common Aucklandia Root (Muxiang) 6g |
| Jianpi Lizhong Decoction | Ginseng (Renshen) 6g, Atractylodes (Fuchaobaizhu) 15g, Fried White Peony Root (Chaobaishao) 15g, Dried Tangerine Peel (Chenpi) 15g, Bran-fried Atractylodes Rhizome (Fuchaocangzhu) 15g, Processed Ginger (Paojiang) 6g, Cimicifuga (Shengma) 10g, Licorice (Gancao) 10g, Nutmeg (Roudoukou) 10g, Stewed Chebula Fruit (Weikezi) 6g |
| Sheng Xian Decoction | Astragalus Root (Huangqi) 30g, Cimicifuga (Shengma) 10g, Platycodon Root (Jiegeng)10g, Common Anemarrhena(Zhimu) 10g, |
